# Microplastic Pollution and Health and Relevance to the Malaysia’s Roadmap to Zero Single-Use Plastics 2018–2030

**DOI:** 10.21315/mjms2020.27.3.1

**Published:** 2020-06-30

**Authors:** Zheng Feei Ma, Yusof Shuaib Ibrahim, Yeong Yeh Lee

**Affiliations:** 1 School of Medical Sciences, Universiti Sains Malaysia, Kubang Kerian, Kelantan, Malaysia; 2 Department of Health and Environmental Sciences, Xi’an Jiaotong-Liverpool University, Suzhou, China; 3 Microplastic Research Interest Group, Faculty of Science and Marine Environment, Universiti Malaysia Terengganu, Terengganu, Malaysia; 4 Gut Research Group, Faculty of Medicine, Universiti Kebangsaan Malaysia, Kuala Lumpur, Malaysia

**Keywords:** microplastics, plastics, health, Malaysia

## Abstract

Microplastic pollution is an emerging environmental and public health threat worldwide including Malaysia. Microplastics are widespread in drinking water, but also food products especially seafood, an important dietary source for the Malaysians. Potential health hazards may be a result of chemicals, physical properties and microbial disturbance associated with microplastic exposure. However, most studies were performed in animals rather than in human. Nevertheless, in recognition of rising threat from microplastics, in 2018, the Malaysia’s Roadmap to Zero Single-use Plastics 2018–2030 has been released. In this editorial, we firstly discussed the potential impact of microplastics on human health, followed by the strategies or limitations highlighted in the Malaysia’s Roadmap.

## Introduction

Microplastics, broadly defined < 5 mm, are plastic particles of different shape, size and polymer composition (1). Recently, there has been extensive media coverage on microplastic pollution including news release from the World Health Organization on the potential environmental health threat from microplastics (2). Single-use plastics and inappropriate plastic waste management are the primary reasons for pollution (3). There are several studies that have investigated the fate and abundance of microplastics, especially in the marine environment (4–5). Polyester, polycarbonate, polypropylene (PP), polyamide, polyvinyl alcohol, polyvinyl chloride (PVC) and polyethylene (PE) are the common polymers in microplastics found in the environment (3–4). Since 1950s, the world plastic production has increased exponentially with current production exceeding 348 million tonnes but only about 9% of plastics are recycled worldwide (6). Production of plastics is forecasted to continue to grow ~3% annually, with Asia being the largest producer (6). Inappropriate plastic waste management is a threat for Malaysia, one of the 10 countries in the world with the biggest threat (7). Of the 0.9 million tons of plastic waste, almost half (0.4 million tons) were inappropriately released into the Malaysian waters (7–8). The most common plastic pollutants found in the Malaysian shore include plastic grocery bags, cigarette buds and plastic bottles (7).

### Are We Eating Microplastics and/or Microplastic-Containing Foods?

According to recent studies, microplastics are widespread in our drinking water, but also found in some food products, especially seafood and salt (9–11). A study by the University of Newcastle reported that an average adult could consume about 5 g of plastic (approximate equivalent to a credit card) weekly from a variety of commonly eaten foods and beverages (12). In addition, there is increasing evidence supporting that microplastics could be ingested by animals and humans via food chains (13). In Malaysia, microplastics are detected in some commercial fish species, which may pose potential health concerns to consumers (14). Aquatic and seafood products are important protein sources and dietary component of many Malaysians with reports of per capita consumption of fish of 58 kg per person (14).

**Figure 1:**
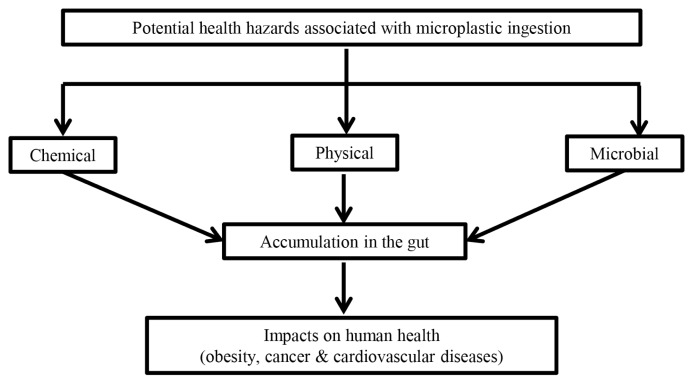
Impacts of microplastic ingestion on health

There are three kinds of potential health hazards associated with microplastic ingestion, and these are chemical, physical particles and microbial pathogens (2) (Figure 1). Firstly, plastics can leach estrogenic-like chemicals (e.g. bisphenol-A or BPA) when exposed to a certain temperature and/or sunlight (ultraviolet radiation) (15). These estrogenic chemicals mimic the actions of naturally occurring estrogens, which subsequently disrupt the endocrine activity with resulting metabolic disorders including obesity and diabetes. In addition, microplastics can absorb and bind harmful additives and monomers including organic pollutants that are present together with microplastics in the environment (15). In mammals, these chemicals found in plastics are associated with increased risk of obesity, some forms of cancers e.g. breast cancer, low sperm count in males and early puberty in females (15). We can postulate that similar adverse consequences are mostly likely found in humans because endocrine system is highly conserved across all vertebrate classes (16). However, confirmatory studies are greatly needed.

Secondly, as a physical particle, after ingestion, some microplastics may pass through the gastrointestinal (GI) tract and are excreted through defecation (17). However, microplastics may potentially accumulate and cause mechanical or physiological disruption to the GI tract and elsewhere. Microplastics may be translocated through blood or lymph to the cardiovascular and respiratory systems causing adverse health consequences (17). For instance, accumulation of microplastics in the circulatory system has been shown to block blood flow and subsequently cause severe damage to the cardiac tissue and its activity (18). In addition, a study has found that inhalable microplastics may also reach the lung alveoli, causing inflammation of the respiratory tract and cardiovascular diseases (19).

Thirdly, microplastics has been shown to induce gut microbiota dysbiosis in fishes, and dysbiosis can interfere with the immune system and trigger life-threatening diseases including infection and death (20). However, the adverse health consequences of short- and long-term microplastic ingestions in humans are not well studied. It is important to be aware that the adverse consequences from microplastic ingestion may depend on the type of microplastics and exposure (i.e. dosedependent) (2).

### The Malaysia’s Roadmap to Zero Single-Use Plastics 2018—2030

In recognition of mounting plastic pollution problem in the country, in 2018, the Malaysian Government has released the Malaysia’s Roadmap to Zero Single-Use Plastics 2018–2030 (Table 1) (7). Several other countries including New Zealand, India, Taiwan and the European Union have introduced similar measures to address plastic pollution. For example, the New Zealand government has banned new single-use plastic bags since 2019. In Thailand, single-use plastic has been banned in major stores beginning 2020 and a complete ban will be implemented in 2021. Likewise, the Indian government is planning to eliminate single-use plastics by 2022. Solving the problem of plastic pollution requires collective efforts from all stakeholders but such regulatory effort from the government is a great step forward (7).

Malaysia has formulated its economic model according to the United Nation’s Sustainable Development Goals (SDGs), one of which is the environmental protection (7). For example, the single-use plastics including plastic straws, wrappers and cutlery should be banned and replaced with more eco-friendly materials. Several states in Malaysia such as Pulau Pinang, Selangor, Kedah and Pahang have also introduced ‘No Plastic Bags Campaign’ or ‘No Plastic Bags Day’ in order to achieve the goal of zero single-use plastics. However, as indicated in the Malaysia’s roadmap, several challenges have been identified (7) and these include: low rate of recycling plastic waste, lack of awareness on sustainable behaviours and habits, lack of integrated waste management, inadequate biodegradability products and high cost of plastic alternatives, and lack of cooperation and enforcement from relevant governmental stakeholders. Therefore, it is vital that each relevant stakeholders, including the federal government, state government, non-government agencies, manufacturers and the general public to work together in order to achieve the goal in 2030 (7). This will then help us move away, especially from the plastic economy and towards a more circular and sustainable ecosystem and a healthier environment.

## Figures and Tables

**Table 1:** Summary of the different phases of the Malaysia’s Roadmap to Zero Single-Use Plastics 2018–2030

Phases	Key actions
1 (2018–2021)	2018 Launch of Roadmap towards Zero Single-Use Plastics 2019 “No straw by default” practice Encouragement of using food containers by customers Pollution charge at RM0.20 for plastic bags Review of existing legal framework on single-use plastics 2020 Launch of a Circular Economy Roadmap (CER) for plastics 2021 Technical workshop for the implementation of CER
2 (2022–2025)	2022 Extension of “No straw by default” practice to non-fixed premises Implementation of CER Extension of minimum pollution charge on plastics bag to non-fixed premises by 2025 2023 Imposition of pollution levy to manufacturers of plastic bags R&D funding on eco-friendly products Implementation of a regional marine debris project Introduction of legal framework on single-use plastics Publication of the mid-term review of the Roadmap
3 (2026–2030)	Expansion scope of compostable and biodegradable products Publication of the implementation report of the Roadmap R&D funding on eco-friendly products
